# Living in uncertainty while a spouse is undergoing a cognitive assessment: Voices of women care partners

**DOI:** 10.1177/14713012221128448

**Published:** 2022-09-21

**Authors:** Ragnhild Hedman, Pernilla Hillerås, Marie Tyrrell

**Affiliations:** Department of Nursing Science, 25548 Sophiahemmet University, Stockholm, Sweden; Red Cross University College, Huddinge, Sweden; Department of Neurobiology, Care Sciences and Society, 97092 Karolinska Institutet, Stockholm, Sweden; Department of Nursing Science, 25548 Sophiahemmet University, Stockholm, Sweden; Department of Neurobiology, Care Sciences and Society, 97092 Karolinska Institutet, Stockholm, Sweden

**Keywords:** caregiver, care partner, cognitive assessment, cognitive impairment, dementia, experience, gender, neuropsychiatric symptoms, uncertainty, wives, women

## Abstract

**Introduction:**

Women often support partners with cognitive symptoms during the assessment process and when they are receiving a cognitive diagnosis. Living with a partner with cognitive symptoms can be stressful; however, there is limited insights into the specific experiences of older women during the assessment process. Previous research indicates that there are gender differences in the experiences of spousal caregiving; however, further research is needed in regard to the experiences of men and women as care partners. Therefore, the aim of the present study was to describe women’s experiences of living with a partner undergoing a cognitive assessment.

**Methods:**

Semi-structured interviews were conducted with seven women when their male partners commenced a cognitive assessment and after the assessment had been completed. The interviews were analysed with abductive content analysis.

**Findings:**

Uncertainty permeated the women’s experiences. Antecedents, attributes and strategies to manage the uncertainty were described.

**Conclusion:**

The participants expressed informational and existential uncertainty when their partner underwent a cognitive assessment. A lack of knowledge regarding the assessment process and cognitive diagnoses, especially mild cognitive impairment, was identified. Further, there was a need to process existential uncertainty evoked by the situation.

## Introduction

Globally, approximately 55 million persons are living with dementia (World Health Organisation, 2021). Risk for developing dementia increases with age, with an estimated eight percent of persons over 65 years, and 50 percent of persons over 90 years developing the disorder (World Health Organisation, 2021). In addition to the cognitive symptoms, neuropsychiatric symptoms, such as anxiety, disrupted sleep pattern and depression, are commonly co-occurring in all stages of cognitive conditions (Van der Linde et al., 2016), posing significant challenges for the persons themselves and their care partners (Fauth & Gibbons, 2014).

In Sweden, care of older persons focuses on providing care in the person’s own home; including persons with extensive support and care needs (Craftman et al., 2018). Most persons with dementia reside in their own homes (National Board of Health and Welfare, 2014), many with support from family members, most commonly spouses (Bökberg et al., 2018). Supporting a person with cognitive impairment is often burdensome (Elmståhl et al., 2018), with care partners experiencing negative impact on their relationships (Holdsworth & McCabe, 2018). Although many couples manage to sustain a sense of couplehood (Bielsten et al., 2018; Hellström et al., 2007), Skaalvik and co-workers (2016) found that supporting a family member with dementia threatened the care partners’ sense of self, leading to an ongoing process of self-preservation and upholding relationships with the family member with dementia. Positive aspects of family caregiving have also been described, related to care partners’ personal achievements, enriched by counselling and formal and informal support received (Lindeza et al., 2020).

Gender differences are known to exist in providing informal care and support for a person with dementia (e.g. Hong & Coogle, 2016), although the research is not consistent (Sharma et al., 2016). Further, care partners are commonly described as a homogeneous group, without consideration to gender and relationship to the person cared for (Bartlett et al., 2018). Ducharme et al. (2011) found that women care partners of persons with Alzheimer’s disease reported more disturbing thoughts concerning their caregiver role, more conflicts in the family and more distress than did men care partners (Ducharme et al., 2011). There are also differences between care partners’ experiences depending on their relationship to the person (Conde-Sala et al., 2010). For example, spouse care partners reported more difficulties in managing disturbing behaviours and problem-solving and less family conflict than adult children care partners (Ducharme et al., 2011). Further, there are differences between the experiences of spouse care partners of persons with younger and late onset of dementia. Care partners of people with late onset dementia felt less prepared and knowledgeable about available services than care partners of people with younger onset dementia (Wawrziczny et al., 2018). While men spouse care partners often described caring as the management of care given by both themselves and others, women tended to perform the care themselves to a wider extent and arrange for other solutions only when their own capacity to care was exhausted (Brown & Chen, 2008). Women spouse care partners have also been described to have poorer mental and physical health and less engagement in health promoting activities than their male counterparts (Gibbons et al., 2014). However, men caring for a spouse with Alzheimer’s disease have also been observed to have more severe sleep disturbance and higher levels of biomarkers of coagulation and inflammation than their female counterparts, with increased risks for cardiovascular disease (Mills et al., 2009). Women care partners are regarded as experiencing a higher burden of care and more depression than their male counterparts (Gibbons et al., 2014; Pillemer et al., 2018), and the presence of neuropsychiatric symptoms in the person with dementia increases the caregiver burden more in women than in men care partners (Bédard et al., 2005). Women care partners’ sense of self was also more affected than that of men (Skaalvik et al., 2016). Walters et al. (2010) interviewed six wives who provided care for their husbands with dementia. The women were concerned about changes in their husbands as persons and their spousal relationships. Further, they reported emotional reactions to their husbands’ impairments and impact of caregiving on their day-to-day lives. A sense of continuity with the past alleviated their adjustment to caregiving (Walters et al., 2010). In a study comparing men and women care partners of a spouse with dementia, the women were more prone to describing their spouses as having changed identity, with marital relationships decreasing in reciprocity and intimacy (Hayes et al., 2009). Hayes and co-workers (2010) also described women observing more early signs of dementia in their spouses, while men tended to downplay and normalize signs. However, women were also more hesitant to bring their observations up with their spouses, most likely to protect their masculine identity.

The Swedish National Guidelines for Dementia Care (National Board of Health and Welfare, 2017) recommend persons with cognitive symptoms to contact primary health care for a cognitive assessment. A cognitive assessment includes: a full medical assessment; interviews with family (if possible); psychological assessments, including assessments of the person’s cognitive performance; and assessment of the person’s function and activity capacity. In the event a person receives a dementia diagnosis, availability of follow-up resources should be in place, meeting the person’s support and care needs (National Board of Health and Welfare, 2017). Persons living with cognitive impairment are encouraged to obtain a timely neurocognitive diagnosis, to enable planning of treatment, care and support for the future (National Board of Health and Welfare, 2017). Family members anticipate that a cognitive assessment can help them gain a greater understanding of the person’s situation and receive validation of symptoms observed (Morgan et al., 2014). Conflicting views can exist within families concerning the necessity of seeking medical care and the impact the cognition impairment has on daily family life (Tyrrell et al., 2021b). Family members often describe struggling to navigate the care system (Prorok et al., 2017). The assessment process can be lengthy and stressful for both the persons with cognitive impairment and the care partners (Prorok et al., 2017; Tyrrell et al., 2021a). In the conversation which discloses a dementia diagnosis, it is critical that the person and their family members are given sufficient information and are not deprived of hope (Poyser & Tickle, 2019).

Family members, especially spouses, are often closely involved in supporting the persons with cognitive symptoms in the assessment process. This has been described as a stressful and daunting period for both the persons with cognitive impairment and the care partners. A limited amount of research is available on experiences of the assessment process from persons with cognitive impairment and family care partner dyads (e.g. Campbell et al., 2016; Walker, et al., 2018). However, to our knowledge, few current studies specifically focus on the experiences of women spouse care partners during the assessment process. More in-depth knowledge in this area could provide a basis for improved caregiver support. Therefore, the aim of the present study was to describe women’s experiences of living with a partner undergoing a cognitive assessment.

## Methods

The present study is associated with two previous studies describing the experiences of 23 persons at the start and end of their medical assessment of cognitive impairment (Tyrrell et al., 2021a; Tyrrell et al., 2021b). The patients’ family members, who accompanied them to the clinic, were also interviewed on both occasions concerning their experiences of supporting the person with cognitive impairment and of the assessment process. For the purpose of the present study, the interviews with wives of the persons with cognitive impairments were selected for analysis. This data has not been previously published.

### Participants

Seven women care partners participated in this study. The women received information about the study from a nurse at a primary health care centre when they accompanied their partners to the clinic for a basic cognitive assessment. Those who agreed to be contacted, were then phoned by one of the researchers (MT) to book the interviews. Inclusion criteria were that the woman’s partner who was commencing a cognitive assessment should be over the age of 65 with a cognitive status of at least 15 points out of 30 on the Mini-Mental State Examination [MMSE] (Folstein et al., 1975). Partners were between 79 and 91 years old and had an MMSE score between 16 and 29. All partners were men. After they had completed the cognitive assessment, three partners were diagnosed with Alzheimer’s disease, one with mixed dementia and three with mild cognitive impairment.

### Data collection

Qualitative, semi-structured interviews (Brinkmann & Kvale, 2018) were conducted with the women on two occasions: at the beginning and end of the partner’s cognitive assessment. The timeframe between the first and second interviews varied between three and 6 months. Interviews were held from October 2017 to December 2019. Four participants were interviewed twice, two were interviewed only at the beginning of the assessment process and one was interviewed only after the completion of the cognitive assessment. A total of 11 interviews were thus conducted. In the first interview, participants were asked if they had influenced their partner’s decision to seek a cognitive assessment, their expectations regarding the cognitive assessment, how their partner’s cognitive impairment had affected their everyday life, if they had observed any neuropsychiatric symptoms in their partners and if they had sufficient support in caring for their partner. In the second interview, we asked if the cognitive assessment had met their expectations and repeated the questions from the first interview about neuropsychiatric symptoms and needs for support. Eight interviews were audio-recorded. During all interviews field notes were taken. On three occasions, the participants were not comfortable with recording the interview. The interviewer then took more extensive notes, which were written up directly after the interviews. As the non-recorded interviews were thoroughly documented and relevant to the aim of the study we decided to include them.

### Ethical considerations

In accordance with the Helsinki Declaration (World Medical Association, 2013) and the ICMJE Recommendations for the protection of research participants (https://www.icmje.org/recommendations/browse/roles-and-responsibilities/protection-of-research-participants.html), written and oral information about the study was provided, and informed consent obtained. Participants were informed that participation was voluntary, that they had a right to withdraw their consent at any time during the data collection phase without stating a reason and that data would be handled confidentially.

### Data analysis

Abductive content analysis (Graneheim et al., 2017), was applied in the analysis. An abductive approach is useful to tie theory and empirical observations together, and develop nursing theory (Råholm, 2010). Initially, two of the researchers (RH, MT) read the interviews and field notes several times to get a grasp of the whole and identify possible themes. In this reading, uncertainty stood out as an overarching theme in the data. A search was carried out for theoretical research on uncertainty, that might deepen the understanding of how the women experienced their situation (Råholm, 2010). Penrod’s (2007) conceptualization of uncertainty was chosen because of its focus on existential uncertainty, which also stood out in our data. A decision was made to continue the analysis deductively, guided by Penrod’s conceptualization. Three categories, antecedents of uncertainty, attributes of uncertainty and strategies to manage uncertainty, were derived from Penrod’s (2007) conceptualization of uncertainty and the data were sorted under these categories. Finally, subcategories were derived from the data under each category. To ensure trustworthiness, each step of the analysis was discussed between the researchers, and alternative ways to interpret and categorise the data was explored until consensus was reached. In the findings, we have strived to present all data relevant to the aim of the study and supply quotes that were representative of the data (Graneheim et al., 2017).

## Findings

The participants’ experiences were permeated by uncertainty. In accordance with Penrod’s (2007) conceptualization, the uncertainty had antecedents and attributes, and the participants had strategies to manage the uncertainty. All participants expressed both uncertainty and strategies to manage to varying degrees. The findings are presented under the categories. Categories and subcategories are compiled in Table 1.

**Table 1.** Overview of the findings.

Categories	Subcategories
Antecedents of uncertainty	• Symptoms and diagnosis
	• The progress of cognitive impairment
	• Co-existent health issues
	• Access to care and support
	• Family finances and everyday life
	• The couples’ relationships
Attributes of uncertainty	• Sense of competence and meaning in caring
	• Disappointment
	• Grief and strain
Strategies to manage uncertainty	• Seeking knowledge and gaining understanding
	• Adaption and trust in available support systems
	• Supporting the partner
	• Engaging in health promotion
	• Managing risks
	• Maintaining normality
	• Utilizing available support

### Antecedents of uncertainty

Participants identified antecedents of uncertainty, including the outcome of their partner’s cognitive assessment, their relationships and thoughts about their future.

#### Uncertainty concerning symptoms and diagnosis

Uncertainty surrounded the assessment process and possible outcomes. Expectations were that a cognitive assessment would provide clarity for the couples’ situation. Some participants were already familiar with Alzheimer’s disease and recognised signs in their partners. They expected the cognitive assessment to verify the diagnosis. In the second interview, one participant whose partner had received an Alzheimer’s diagnosis, was uncertain and deliberated about if it had been right, in hindsight, to seek an assessment. The participant described how her partner (aged 81 with Alzheimer’s disease) would now have to live with the ‘dementia label’:

Sometimes I wonder if it was right to make that diagnosis. Maybe it would have been better not to know. At the same time, it was good as you can still slow it down… Maybe it would be better not to know at all?

Several participants whose partners had received a diagnosis (mild cognitive impairment or Alzheimer’s disease) were uncertain about the accuracy of the diagnosis and the quality of the cognitive assessment. For one, the assessment had not been carried out in full as her partner had been unable to complete the routine testing due to impaired vision. Despite this, an Alzheimer’s diagnosis had been determined, which the couple appeared unaware of. The couple were waiting to receive information about a follow-up. Another participant was surprised that her partner had been diagnosed with mild cognitive impairment and not Alzheimer’s disease. In her opinion her partner had great difficulties in his daily life, which was not reflected in his cognitive test results. Yet another participant, whose partner had been diagnosed with mild cognitive impairment, said that her partner (aged 82) had received the diagnosis ‘not Alzheimer’s’. She questioned the quality of the cognitive assessment, as the couple experienced that the psychologist had fallen asleep during the neuropsychological testing.

For one participant, spouse to a man aged 78, the diagnosis of mild cognitive impairment implied an end to uncertainty: ‘This diagnosis does not imply a further deterioration… It can happen to all older people. So, I think… this was very positive that it was not one of the pathological memory diseases if I may call them so.’

#### Uncertainty about future progress of cognitive impairment

Uncertainty also applied to the future progress of symptoms, possible treating options and their own capacity to cope. Participants were worried that their partners’ cognitive impairments would increase and that this would have negative impact on their relationship and everyday life. Concerns were expressed that partners would become increasingly more dependent on them and lose their sense of self and perception of reality. A spouse to a man aged 91 with Alzheimer’s disease described the situation as:

I clearly have worries that it will get worse. We have friends where one partner does not recognise the other; that is a terrifying scenario… The biggest problem for me is the anxiety and worry about the future… That we could not continue our life as it is. We have a very good life; that would be the greatest loss.

Participants hoped that their partners would be prescribed medication that would halt, invert, or delay the progress of cognitive impairment. This stood out as a strong motivator for seeking a cognitive assessment. Further, they expressed uncertainty about possibilities for delaying the disease progression by cognitive training and a healthy lifestyle. ‘If you can somehow help him… if this is some type of process towards the worst…, can it be slowed down and, in that case, what can you do and how can you train the memory back?’ (Spouse to a man aged 81 with Alzheimer’s disease).

Some participants also expressed uncertainty concerning their ability to care, which depended on themselves keeping healthy and able to cope when cognitive impairment deteriorated.

#### Uncertainty concerning co-existent health issues

Several partners with cognitive impairment were living with other health issues contributing to the uncertainty of participants. ‘The participant [a spouse to a man aged 91 with Alzheimer’s disease] looked tired… She was close to tears recounting her partner’s recent hospitalisation, thinking he would not survive’ (field note).

Partners’ pre-existing health conditions were sometimes more challenging for participants than the cognitive impairment. Multiple health issues entailed more health care contacts which presented a challenge and caused exhaustion. There were also increased risks, for example of fall, which added to the participants’ uncertainty.

#### Uncertainty about the availability and quality of care and support

The access to quality care and support was a source of uncertainty. As previously mentioned, some participants were not convinced their partners had been correctly assessed and diagnosed regarding their cognitive impairment. Further, participants were uncertain about possibilities of their partners being prescribed medication, the effect of medication and other post-diagnostic care and support. They were also uncertain about what a cognitive diagnosis entailed. A spouse to a man aged 91 with Alzheimer’s disease stated:

It was difficult to know what to expect. The goal is to find suitable medication… this I think is very important… The assessment is not completed as my partner could not answer certain questions because he cannot see, and this has not been taken into consideration … one should be able to see and draw a clock and such things… it (the process) is flawed.

Several participants were uncertain about the quality of home care services and stated that they lacked continuity and choice: ‘It didn’t work out as the level of service offered were pre-determined, he could only get a shower once a week’ (spouse to man aged 81 with Alzheimer’s disease). One participant also expressed uncertainty about the competence of the community care manager who conducted a need assessment and appeared young and inexperienced. Uncertainties were also present surrounding the possibilities of receiving economical support for being a family caregiver and about the reliability of services offered by the municipality.

#### Uncertainty about family finances and everyday life

Uncertainty also concerned the management of the couples’ daily lives. Some participants found it difficult to leave their partners alone at home, as they became anxious when their spouses were not present. A participant described that her partner’s memory problems had caused them to miss appointments and other engagements. Travelling and renovations of the home had become more difficult, as they were stressful for the partner. Further, one participant expressed uncertainty about following through with plans to move and whether it would be best to remain in the environment that was well known to her partner or not. One participant also described how her partner was losing his ability to manage his personal finances and tax declarations, which caused great uncertainty for her about the best way to handle the situation, as it was regarded as a sensitive issue.

#### Uncertainty about the impact on the couples’ relationships

Participants described increased uncertainty in their relationships with their partners. There were more misunderstandings in their communication. Their partners had become more irritable and sensitive to perceived criticism. One participant described how her partner was previously very calm and solved all kinds of situations. He was now easily worried and stressed as he lacked total control of situations. She was worried about how this affected him (a man aged 81 with Alzheimer’s disease) and their wellbeing as a couple.

Sometimes I think that through anxiety a person disappears piece by piece and does not have a physical presence… We have been together for 20 years and have done everything together, no matter what… I had a relative with dementia (laughs) who moved into a care home. Her husband came to visit, and she happily announced she had married her neighbour… it is terrible to lose sight of reality.

### Attributes of uncertainty

Participants experienced uncertainty in various way. Most commonly they declared that they were able to manage. However, they also spoke about feelings of grief, disappointment and stress related to uncertainty.

#### Sense of competence and meaning in caring

Some participants said that they had knowledge about Alzheimer’s disease which decreased their uncertainty and that caring was meaningful to them. They saw it as a natural part of their marriage to help one another and described that they had a strong relationship and were determined to maintain it for as long as possible.

Nothing negative has come out of this. We are at the beginning of these symptoms I would say… the fact that we are always together and do things together. I help out to the extent that he needs help, I have done so for the past 12 years since he lost his sight… we are a team that go arm in arm (spouse to a man aged 91 with Alzheimer’s disease).

#### Disappointment

Disappointment occurred when participants felt that the cognitive assessment had failed to provide a reliable cognitive diagnosis. The apparent lack of medical follow-up after a mild cognitive impairment diagnosis was also a source of disappointment. A spouse to a man aged 87 with mild cognitive impairment described the situation: ‘The assessment was not what I envisaged… no follow-up which we would like’. Further, one participant (spouse to man aged 84 with mixed dementia) expressed disappointment with the financial support she, as a family caregiver received.

I applied for family support and received 2000 SEK per month. I have a friend who got more so I returned to the municipality and specified what I do for my husband. I had previously written that I do everything but needed to specify what I actually do … I save the state a lot.

Disappointment was also experienced when participants felt unfairly treated by their partners, as when being blamed for mistakes which were due to the presence of cognitive impairment.

#### Grief and strain

Participants expressed grief, caused by the present and expected change in the partners’ ways of being and the couples’ ways of lives, also expressed feeling sorry for their partners. A spouse to a man aged 91 with Alzheimer’s disease said: ‘There’s a great change in our lives compared to how it was… a lot has happened… it is sad.’ The spouse also added: ‘I feel sad when he forgets what we talked about a half an hour ago… I can get upset; I feel sorry for him.’ The uncertain situation with the debut of cognitive symptoms and the cognitive assessment caused strain for participants that was sometimes accelerated by other stressful life events. The management of their partners’ health issues was sometimes described as exhausting. Partners’ increased anxiety and dependence on participants was described as strenuous: ‘It can get annoying … Your worries can be stressful for me (talking to partner)’ (spouse to a man aged 81 with Alzheimer’s disease).

### Strategies to manage uncertainty

With a view to decrease uncertainty, a range of approaches were used.

#### Seeking knowledge and gaining understanding

When cognitive decline became apparent for the participants, they encouraged their partners to mention their symptoms to their GPs. Participants expressed the importance of having a greater understanding of the situation to be able to make the best of it. The diagnosis made it easier for participants to understand the nature of their partners’ problems, and how to support them. One participant (spouse to man aged 81 with Alzheimer’s disease) described that she had changed her behaviour towards her partner and was more patient with him, after his diagnosis.

My attitude has changed, I try to have a greater understanding and treat you (speaking to partner) better and not to get so annoyed… Not that I was very annoyed before, but I have a different kind of understanding now, like when you have asked me two or three times the same thing.

Learning about the diagnosis had also made it easier to plan for the future, for example, making decisions about travelling and moving.

#### Adaption and trust in available support systems

Participants had adapted their everyday life and expressed trust in the available support systems. They had taken over various chores that their partners had previously handled and acknowledged their new situation when planning activities, such as visiting the theatre, travelling, renovating and moving. Participants were satisfied with the care that they had received so far concerning their partner’s cognitive impairment and were grateful that the partner (aged 81 with Alzheimer’s disease) had received medication that would ‘stop’ the process of cognitive decline. ‘One is so grateful that there is some kind of braking mechanism that it doesn’t go any further… then I dread to think of what might have happened if we had not sought help and got this’.

Some also referred to previous experiences with health care and social services and said that when you really need it, help is available. Trust in support also included trust in informal support when needed.

#### Supporting the partner

Uncertainty was managed by supporting the partner. In some cases, participants offered extensive support to their partners and were totally relied upon. Support consisted of providing reminders and reality orientation, adjusting activities, managing the partner’s health care appointments, accompanying, guiding and taking over household chores and driving. A spouse to a man aged 82 with mild cognitive impairment stated that she had started to support her husband with appointments and dental care visits. She said she was happy to do this as they were married for 50 years and got on well. Participants also assisted their partners’ social and intellectual involvement by explaining things that they did not grasp, for example, when listening to the news or going to the theatre or cinema.

#### Engaging in health promotion

Uncertainty was also mastered by health promotive activities together with their partner. Finding enjoyment and keeping physically and mentally active was important, for example walking and doing crossword puzzles. One couple (including man aged 81 with Alzheimer’s disease) had also started to take dietary supplements.

I bought vitamin B-complex tablets, the type that boost your immune system, for the two of us. We have even increased the time spent on solving crosswords…to keep the brain active… Yesterday we went out for a walk, and he took an extra round when I came home to fix the potatoes.

Further, the medication prescribed for the partner’s Alzheimer’s disease was described as vital to promote the partner’s cognitive health and delay deterioration.

#### Managing risks

Managing uncertainty also included managing risks and planning for adverse events. One participant described how she had instructed her partner (aged 84 with mixed dementia) to seek assistance from their neighbour in case she would fall on the floor: ‘We have a plan of action if I should fall; he will take the elevator up to our neighbour’. The neighbour was aware of the couple’s situation and had helped them to manage crises, such as when one of them could not get out of the bathtub, and on another occasion when the participant was locked out.

#### Maintaining normality

One way to concur uncertainty was to maintain their previous lifestyle: ‘We feel that we should continue as we did, like going to art exhibitions, going to the theatre’. (Spouse to a man aged 84 with mixed dementia). Although some adjustments were often required to meet the partner’s needs, being able to continue their previous way of life was regarded as important: ‘We try to go out every day and do something fun. If we go to the cinema we must sit in the front row.’

#### Utilizing available support

Support was provided from family, neighbours, friends and from society. Support from society included transportation service, home care services, day care and support groups. Several were utilizing transportation service and considering the possibility of adult day care. One participant had recently accepted home care services and chosen a small company for the sake of staff continuity. Further, one participant was seeking support for her partner (aged 84 with mild cognitive impairment) to manage his personal finances: ‘My husband needs help organising his finances… we need to connect with someone… it’s about practical things’. Participants were also interested in accessing support groups.

## Discussion

The purpose of this study was to describe women’s experiences of living with a partner undergoing a cognitive assessment. The core finding was that a sense of uncertainty permeated the women’s experiences. According to Penrod (2007, p. 664, Figure 2), ‘Uncertainty is a perception of doubt or not knowing that is brought about by cognitive and pre-cognitive ways of knowing’. Antecedents of uncertainty were related to several aspects of living with a partner with cognitive impairment, including a lack of knowledge and existential concerns. This was in line with Penrod’s (2007) and others’ (e.g. Dwan & Willig, 2021; Han et al., 2011) conceptualisations, describing that health-related uncertainty has both informational and existential antecedents. Informational uncertainty can potentially be mediated by improved patient education. Existential uncertainty, however, concerns for example questions about meaning and identity, being in the world and mortality (Dwan & Willig, 2021). It cannot be solved with information, rather the person needs to come to terms with the uncertainty (Penrod, 2007). Health care professionals can support this process by listening and supporting their sense of confidence and control. The attributes of uncertainty, in our findings, were a sense of competence and meaning, disappointment and grief and strain. According to Penrod (2007), a sense of control and confidence (or the lack thereof) determines types of uncertainty. For example, the combination of low control and low confidence fosters overwhelming uncertainty, which resembles our subcategory grief and strain. High levels of control and confidence results in minimal uncertainty, which resembles our subcategory sense of competence and meaning in caring. To our knowledge, disappointment has not been previously described as an attribute of uncertainty. However, Zeelenberg et al. (2000) described that disappointment can provoke feelings of powerlessness and loss of control. As a lack of control increases uncertainty, it seems likely that this could explain how the concepts of uncertainty and disappointment are related. Uncertainty was managed by applying various strategies, ranging from seeking knowledge to maintaining normality and engaging in health promotive activities. This too resonates well with Penrod’s conceptualisation, which describes how strategies to manage uncertainty were focused on (re-)gaining control and confidence, gathering information, focussing on the present and establishing new ways of being.

Uncertainty has been frequently described in research concerning the experiences of persons with cognitive impairment and their care partners (e.g. Campbell et al., 2016; Nilsson & Olaison, 2019; Stokes et al., 2015; Van Wijngaarden et al, 2018). However, few studies have applied a theoretical framework. Unson and co-workers (2015), applying Mishel’s (2014) theory of illness uncertainty, found that care partners of people with dementia, mainly adult children, experienced stress similar to that of patients with life-altering illness. Further, none of these studies have applied a gender perspective on uncertainty. This supports Bartlett’s et al. (2018) observation that research about cognitive impairment, even that which has focused on matters that are central to the identity of people who are living with the condition, often appears to be gender blind. Previous studies about differences between men and women care partners of spouses with cognitive impairments have shown that men tend to be less observant of emerging symptoms but more active in seeking help once they become aware that there is something wrong (Hayes et al., 2010), less concerned about the change in identity in their spouse and its effect on the marital relationship (e.g. Hayes et al., 2009), and more able to control disturbing thoughts (Ducharme et al., 2011). These are qualities that may mediate the sense of uncertainty, resulting in lower levels of uncertainty in men than in women. Research on gender differences in family caregiving generally shows that women spend more time caring, perform more demanding care, experience more role conflicts and role strain, feel more obliged to care and experience higher burden and negative impacts on their own health from caregiving than men. This research, however, is inconsistent, with other studies showing no or small gender differences (Sharma et al., 2016). Nevertheless, it seems likely to assume that women may feel less in control of their caregiver role, and thus experience higher uncertainty. Prolonged periods of caregiving and perceived lack of support, which are often described as features of women’s caregiving (Sharma et al., 2016) were also associated with increased levels of uncertainty in caregivers of people who receive palliative care (Arias-Rojas et al., 2019). This finding further supports the likelihood of women being more exposed to uncertainty in caregiving. Future studies should focus on the experiences of male care partners while their partners are undergoing a cognitive assessment, and on gender differences in the experiences of uncertainty.

The availability and access to quality care and support were highlighted by participants as a source of uncertainty. Several partners had received a mild cognitive impairment diagnosis, which was deemed an abstract diagnosis and for some not representative of their partners’ condition. Receiving a mild cognitive impairment diagnosis raised questions regarding the reliability of the assessment, fuelling feelings of uncertainty. The quality of the assessment was also questioned regarding professionalism of staff and rigidity of the assessment, which did not accommodate a person’s varying levels of function and limitations. Similarly, Samsi et al. (2014) identified how a standard cognitive assessment often failed to place the person with cognitive impairment in focus. Researchers (e.g. Schermer & Richard, 2019) argue that a diagnosis of ‘preclinicalAlzheimer’s disease’ may cause uncertainty and anxiety in patients and question the utility of such diagnostics in the absence of treatment. However, our participants had already noticed signs of cognitive impairment and were searching for answers. In such cases, improved information and follow-up after the diagnosis might be more useful to the patients and their families than withholding a cognitive assessment.

For partners with mild cognitive impairment, it was unclear what support services and care were available. For others, home care appeared to be standardised and not tailored to individual needs. According to Frederiksen et al. (2021), it is important that post-diagnosis follow-up for persons with mild cognitive impairment is individualised, creating a care plan with the person and identifying their needs. Meeting information needs can empower the spouses and alleviate uncertainty. Soong et al. (2020) identified four categories of information needs of persons and family members: available health care services (most common), disease, providing care and self-care. Types of information needs varied pending the severity of the person’s cognitive impairment and the status of the person. It is also important that support of persons with cognitive impairments and their families is gender sensitive and based on knowledge about differences in the needs and experiences of men and women.

### Limitations

There are limitations to this study. To comply with the participants wishes, their partners were partly present during all interviews and some interviews were not audio-recorded. The participants appeared to speak freely despite their partners’ presence; however, this may have affected the data. Immediate transcription of the extensive field notes made during the non-recorded interviews enhanced the chances of accurate documentation of the interview contents. In the findings, we have avoided to quote from the non-recorded interviews because such quotes would not be verbatim. Instead, we chose to describe in our own words what was said in the non-recorded interviews. The choice to apply Penrod’s conceptualization of uncertainty (2007) early in the analysis may have overshadowed the possibility of other interpretations of the data. However, as Råholm (2010) stated, qualitative research is not about finding the absolute truth, rather it aims to enhance a more profound understanding of the phenomena under study. Because interpretation occurs in a dialogue between the researchers and the text (Graneheim et al., 2017), it is quite possible that other researchers would have interpreted the data differently. Throughout the paper, we have strived to provide sufficient information to assess the credibility of the findings.

## Conclusion

Female spouses’ experiences of their partners’ cognitive assessment were overshadowed by uncertainty. Uncertainty experienced had both informational and existential dimensions and was similar to that which has been described in other groups of patients and care partners, living with severe and life limiting conditions. Support directed to spousal care partners needs to be gender sensitive and focused on alleviating informational and existential uncertainty in relation to the different types of cognitive diagnoses. Receiving a mild cognitive impairment diagnosis was a significant source of uncertainty, calling for special attention from professionals to provide the necessary information and support.

## References

[bibr1-14713012221128448] Arias-Rojas M. Carreño-Moreno S. Posada-López C. (2019). Uncertainty in illness in family caregivers of palliative care patients and associated factors. Revista Latino-Americana de Enfermagem, 27, e3200. 10.1590/1518-8345.3185.3200 31618393 PMC6792341

[bibr2-14713012221128448] Bartlett R. Gjernes T. Lotherington A. T. Obstefelder A. (2018). Gender, citizenship and dementia care: A scoping review of studies to inform policy and future research. Health & Social Care in the Community, 26(1), 14–26. 10.1111/hsc.12340 26990695

[bibr3-14713012221128448] Bédard M. Kuzik R. Chambers L. Molloy D. W. Dubois S. Lever J. A. (2005). Understanding burden differences between men and women caregivers: The contribution of care-recipient problem behaviors. International psychogeriatrics, 17(1), 99–118. 10.1017/s1041610204000857 15945595

[bibr4-14713012221128448] Bielsten T. Lasrado R. Keady J. Kullberg A. Hellström I. (2018). Living life and doing things together: Collaborative research with couples where one partner has at diagnosis of dementia. Qualitative Health Research, 28(11), 1719–1734. 10.1177/1049732318786944 30033851

[bibr5-14713012221128448] Bökberg C. Ahlström G. Karlsson S. (2018). Utilisation of formal and informal care and services at home among persons with dementia: A cross-sectional study. Scandinavian Journal of Caring Sciences, 32(2), 843–851. 10.1111/scs.12515 28869661

[bibr6-14713012221128448] Brinkmann S. Kvale S. (2018). Doing interviews (2d ed.). Sage.

[bibr7-14713012221128448] Brown J. Chen S. L. (2008). Help-seeking patterns of older spousal of older adults with dementia. Issues in Mental Health Nursing, 29(8), 839–852. 10.1080/01612840802182854 18649210

[bibr8-14713012221128448] Campbell S. Manthorpe J. Samsi K. Abley C. Robinson L. Watts S. Bond J. Keady J. (2016). Living with uncertainty: Mapping the transition from pre-diagnosis to a diagnosis of dementia. Journal of Aging Studies, 37, 40–47. 10.1016/j.jaging.2016.03.001 27131277

[bibr9-14713012221128448] Conde-Sala J. L. Garre-Olmo J. Turró-Garriga O. Vilalta-Franch J. López-Pousa S. (2010). Differential features of burden between spouse and adult-child of patients with Alzheimer's disease: An exploratory comparative design. International Journal of Nursing Studies, 47(10), 1262–1273. 10.1016/j.ijnurstu.2010.03.001 20374966

[bibr10-14713012221128448] Craftman Å. G. Grundberg Å. Westerbotn M. (2018). Experiences of home care assistants providing social care to older people: A context in transition. International Journal of Older People Nursing, 13(4), e12207. 10.1111/opn.12207 30063125

[bibr11-14713012221128448] Ducharme F. Lévesque L. Lachance L. Kergoat M. J. Coulombe R. (2011). Challenges associated with transition to caregiver role following diagnostic disclosure of alzheimer disease: A descriptive study. International journal of nursing studies, 48(9), 1109–1119. 10.1016/j.ijnurstu.2011.02.011 21376317

[bibr12-14713012221128448] Dwan C. Willig C. (2021). Existential uncertainty in health care: A concept analysis. Journal of Evaluation in Clinical Practice, 27(3), 562–570. 10.1111/jep.13536 33474766

[bibr13-14713012221128448] Elmståhl S. Dahlrup B. Ekström H. Nordell E. (2018). The association between medical diagnosis and caregiver burden: A cross-sectional study of recipients of informal support and caregivers from the general population study ‘good aging in skåne’, Sweden. Aging Clinical and Experimental Research, 30(9), 1023–1032. 10.1007/s40520-017-0870-0 29236217 PMC6096871

[bibr14-14713012221128448] Fauth E. B. Gibbons A. (2014). Which behavioral and psychological symptoms of dementia are the most problematic? Variability by prevalence, intensity, distress ratings, and associations with carer depressive symptoms. International Journal of Geriatric Psychiatry, 29(3), 263–271. 10.1002/gps.4002 23846797

[bibr15-14713012221128448] Folstein M. F. Folstein S. E. McHugh P. R. (1975). Mini-mental state. A practical method for grading the cognitive state of patients for the clinician. Journal of psychiatric research, 12(3), 189–198. 10.1016/0022-3956(75)90026-6 1202204

[bibr16-14713012221128448] Frederiksen K. S. Nielsen T. R. Winblad B. Schmidt R. Kramberger M. G. Jones R. W. Hort J. Grimmer T. Georges J. Frölich L. Engelborghs S. Dubois B. Waldemar G. (2021). European academy of neurology/European alzheimer’s disease consortium position statement on diagnostic disclosure, biomarker counseling, and management of patients with mild cognitive impairment. European Journal of Neurology, 28(7), 2147–2155. 10.1111/ene.14668 33368924 PMC8246881

[bibr17-14713012221128448] Gibbons C. Creese J. Tran M. Brazil K. Chambers L. Weaver B. Bédard M. (2014). The psychological and health consequences of caring for a spouse with dementia: A critical comparison of husbands and wives. Journal of Women & Aging, 26(1), 3–21. 10.1080/08952841.2014.854571 24483280

[bibr18-14713012221128448] Graneheim U. H. Lindgren B. M. Lundman B. (2017). Methodological challenges in qualitative content analysis: A discussion paper. Nurse education today, 56, 29–34. 10.1016/j.nedt.2017.06.002 28651100

[bibr19-14713012221128448] Han P. K. J. Klein W. M. P. Arora N. K. (2011). Varieties of uncertainty in health care: A conceptual taxonomy. Medical Decision Making, 31(6), 828–838. 10.1177/0272989X10393976 22067431 PMC3146626

[bibr20-14713012221128448] Hayes J. Boylstein C. Zimmerman M. K. (2009). Living and loving with dementia: Negotiating spousal and caregiver identity through narrative. Journal of Aging Studies, 23(1), 48–59. 10.1016/j.jaging.2007.09.002

[bibr21-14713012221128448] Hayes J. Zimmerman M. K. Boylstein C. (2010). Responding to symptoms of alzheimer’s disease: Husbands, wives, and the gendered dynamics of recognition and disclosure. Qualitative Health Research, 20(8), 1101–1115. 10.1177/1049732310369559 20448273

[bibr22-14713012221128448] Hellström I. Nolan M. Lundh U. (2007). Sustaining ’couplehood’. Spouses’ strategies for living positively with dementia. Dementia: The International Journal of Social Research and Practice, 6(3), 383–409. 10.1177/1471301207081571

[bibr23-14713012221128448] Holdsworth K. McCabe M. (2018). The impact of dementia on relationships, intimacy, and sexuality in later life couples: An integrative analysis of existing literature. Clinical Gerontologist, 41(1), 3–19. 10.1080/07317115.2017.1380102 29161218

[bibr24-14713012221128448] Hong S. Coogle C. L. (2016). Spousal caregiving for partners with dementia: A deductive literature review testing calasanti’s gendered view of care work. Journal of Applied Gerontology, 35(7), 759–787. 10.1177/0733464814542246 25037154

[bibr25-14713012221128448] Lindeza P. Rodrigues M. Costa J. Guerreiro M. Rosa M. M. (2020). Impact of dementia on informal care: A systematic review of family caregivers' perceptions. BMJ supportive & palliative care. 10.1136/bmjspcare-2020-002242 33055092

[bibr52-14713012221128448] Mills P. J. Ancoli-Israel S. von Känel R. Mausbach B. T. Aschbacher K. Patterson T. L. Ziegler M. G. Dimsdale J. E. Grant I. (2009). Effects of gender and dementia severity on Alzheimer's disease caregivers' sleep and biomarkers of coagulation and inflammation. Brain, Behavior, and Immunity, 23(5), 605–610. 10.1016/j.bbi.2008.09.014 18930805 PMC2757046

[bibr26-14713012221128448] Mishel M. H. (2014). Theories of uncertainty in illness. In Smith M. J. Liehr P. R. (Eds.), Middle range theories for nursing (3rd ed.). Springer, pp. 63–93.

[bibr27-14713012221128448] Morgan D. G. Walls-Ingram S. Cammer A. O'Connell M. E. Crossley M. Dal Bello-Haas V. Forbes D. Innes A. Kirk A. Stewart N. (2014). Informal caregivers’ hopes and expectations of a referral to a memory clinic. Social Science & Medicine, 102, 111–118. 10.1016/j.socscimed.2013.11.023 24565148

[bibr28-14713012221128448] National Board of Health and Welfare. (2017). Nationella riktlinjer för vård och omsorg vid demenssjukdom: Stöd för styrning och ledning. Socialstyrelsen. https://www.socialstyrelsen.se/globalassets/sharepoint-dokument/artikelkatalog/nationella-riktlinjer/2017-12-2.pdf

[bibr29-14713012221128448] National Board of Health and Welfare. (2014). Demenssjukdomarnas samhällskostnader i Sverige 2012. Socialstyrelsen. https://docplayer.se/10737546-Demenssjukdomarnas-samhallskostnader-i-sverige-2012.html

[bibr30-14713012221128448] Nilsson E. Olaison A. (2019). What is yet to come? Couples living with dementia orienting themselves towards an uncertain future. Qualitative Social Work, 18(3), 475–492. 10.1177/1473325017743104

[bibr31-14713012221128448] Penrod J. (2007). Living with uncertainty: Concept advancement. Journal of Advanced Nursing, 57(6), 658–667. 10.1111/j.1365-2648.2006.04008.x 17346325

[bibr32-14713012221128448] Pillemer S. Davis J. Tremont G. (2018). Gender effects on components of burden and depression among dementia caregivers. Aging & Mental Health, 22(9), 1156–1161. 10.1080/13607863.2017.1337718 28604059 PMC6107424

[bibr33-14713012221128448] Poyser C. A. Tickle A. (2019). Exploring the experience of the disclosure of a dementia diagnosis from a clinician, patient and carer perspective: A systematic review and meta-ethnographic synthesis. Aging & Mental Health, 23(12), 1605–1615. 10.1080/13607863.2018.1506747 30430858

[bibr34-14713012221128448] Prorok J. C. Hussain M. Horgan S. Seitz D. P. (2017). ‘I shouldn’t have had to push and fight’: Health care experiences of persons with dementia and their caregivers in primary care. Aging & Mental Health, 21(8), 797–804. 10.1080/13607863.2016.1159280 26982159

[bibr35-14713012221128448] Råholm M. B. (2010). Abductive reasoning and the formation of scientific knowledge within nursing research. Nursing Philosophy: An International Journal for Healthcare Professionals, 11(4), 260–270. 10.1111/j.1466-769X.2010.00457.x 20840137

[bibr36-14713012221128448] Samsi K. Abley C. Campbell S. Keady J. Manthorpe J. Robinson L. Watts S. Bond J. (2014). Negotiating a labyrinth: Experiences of assessment and diagnostic journey in cognitive impairment and dementia. International Journal of Geriatric Psychiatry, 29(1), 58–67. 10.1002/gps.3969 23625551

[bibr37-14713012221128448] Schermer M. H. N. Richard E. (2019). On the reconceptualization of Alzheimer’s disease. Bioethics, 33(1), 138–145. 10.1111/bioe.12516 30303259 PMC6585806

[bibr38-14713012221128448] Sharma N. Chakrabarti S. Grover S. (2016). Gender differences in caregiving among family - Caregivers of people with mental illnesses. World Journal of Psychiatry, 6(1), 7–17. 10.5498/wjp.v6.i1.7 27014594 PMC4804270

[bibr39-14713012221128448] Skaalvik M. W. Norberg A. Normann K. Fjelltun A.-M. Asplund K. (2016). The experience of self and threats to sense of self among relatives caring for people with Alzheimer’s disease. Dementia, 15(4), 467–480. 10.1177/1471301214523438 24535820

[bibr40-14713012221128448] Soong A. Au S. T. Kyaw B. M. Theng Y. L. Tudor Car L. (2020). Information needs and information seeking behaviour of people with dementia and their non-professional caregivers: A scoping review. BMC geriatrics, 20(1), 61. 10.1186/s12877-020-1454-y 32059648 PMC7023704

[bibr41-14713012221128448] Stokes L. Combes H. Stokes G. (2015). The dementia diagnosis: A literature review of information, understanding, and attributions. Psychogeriatrics, 15(3), 218–225. 10.1111/psyg.12095 25515569

[bibr42-14713012221128448] Tyrrell M. Hedman R. Fossum B. Skovdahl K. Religa D. Hillerås P. (2021a). Feeling valued versus abandoned: Voices of persons who have completed a cognitive assessment. International Journal of Older People Nursing, 16(6), e12403. 10.1111/opn.12403 34231964

[bibr43-14713012221128448] Tyrrell M. Religa D. Fossum B. Hedman R. Skovdahl K. Hillerås P. (2021b). Embarking on a memory assessment voices of older persons living with memory impairment. Dementia, 20(2), 717–733. 10.1177/1471301220910637 32188280 PMC7983336

[bibr44-14713012221128448] Unson C. Flynn D. Glendon M. A. Haymes E. Sancho D. (2015). Dementia and caregiver stress: An application of the reconceptualized uncertainty in illness theory. Issues in Mental Health Nursing, 36(6), 439–446. 10.3109/01612840.2014.993052 26241570

[bibr45-14713012221128448] Van der Linde R. M. Dening T. Stephan B. C. Prina A. M. Evans E. Brayne C. (2016). Longitudinal course of behavioural and psychological symptoms of dementia: Systematic review. The British Journal of Psychiatry: The Journal of Mental Science, 209(5), 366–377. 10.1192/bjp.bp.114.148403 27491532 PMC5100633

[bibr46-14713012221128448] Van Wijngaarden E. van der Wedden H. Henning Z. Komen R. The A. M. (2018). Entangled in uncertainty: The experience of living with dementia from the perspective of family caregivers. Plos One, 13(6), 1–21. 10.1371/journal.pone.0198034 PMC5999274 29897922

[bibr47-14713012221128448] Walker R. Ratcliffe J. White A. Visvanathan R. (2018). Dementia assessment services: What are the perceptions of older people? Australasia Journal of Ageing, 37(1), 43–47. 10.1111/ajag.12455 29024346

[bibr53-14713012221128448] Walters A. H. Oyebode J. R. Riley G. A. (2010). The dynamics of continuity and discontinuity for women caring for a spouse with dementia. Dementia, 9(2), 169–189. 10.1177/1471301209354027

[bibr48-14713012221128448] Wawrziczny E. Berna G. Ducharme F. Kergoat M. J. Pasquier F. Antoine P. (2018). Characteristics of the spouse caregiving experience: Comparison between early- and late-onset dementia. Aging & Mental Health, 22(9), 1207–1215. 10.1080/13607863.2017.1339777 28631510

[bibr49-14713012221128448] World Medical Association. (2013). WMA declaration of Helsinki: Ethical principles for medical research involving human subjects. https://www.wma.net/policies-post/wma-declaration-of-helsinki-ethical-principles-for-medical-research-involving-human-subjects/10.1001/jama.2013.281053 24141714

[bibr50-14713012221128448] World Health Organisation. (2021). Dementia: Key facts. https://www.who.int/news-room/fact-sheets/detail/dementia

[bibr51-14713012221128448] Zeelenberg M. van Dijk W. W. Manstead A. S. R. van der Pligt J. (2000). On bad decisions and disconfirmed expectancies: The psychology of regret and disappointment. Cognition & Emotion, 14(4), 521–541. 10.1080/026999300402781

